# Targeted Therapies in Type II Endometrial Cancers: Too Little, but Not Too Late

**DOI:** 10.3390/ijms19082380

**Published:** 2018-08-13

**Authors:** Michiel Remmerie, Veerle Janssens

**Affiliations:** 1 Laboratory of Protein Phosphorylation & Proteomics, Department of Cellular & Molecular Medicine, University of Leuven (KU Leuven), B-3000 Leuven, Belgium; michiel.remmerie@kuleuven.be; 2 Leuven Cancer Institute (LKI), B-3000 Leuven, Belgium

**Keywords:** endometrial cancer, type II endometrial carcinoma, targeted therapy, kinase inhibitor, molecular marker, protein kinase, protein phosphatase, PP2A, *PPP2R1A*, SMAP

## Abstract

Type II endometrial carcinomas (ECs) are responsible for most endometrial cancer-related deaths due to their aggressive nature, late stage detection and high tolerance for standard therapies. However, there are no targeted therapies for type II ECs, and they are still treated the same way as the clinically indolent and easily treatable type I ECs. Therefore, type II ECs are in need of new treatment options. More recently, molecular analysis of endometrial cancer revealed phosphorylation-dependent oncogenic signalling in the phosphatidylinositol-4,5-bisphosphate 3-kinase (PI3K) and mitogen-activated protein kinase (MAPK) pathways to be most frequently altered in type II ECs. Consequently, clinical trials tested pharmacologic kinase inhibitors targeting these pathways, although mostly with rather disappointing results. In this review, we highlight the most common genetic alterations in type II ECs. Additionally, we reason why most clinical trials for ECs using targeted kinase inhibitors had unsatisfying results and what should be changed in future clinical trial setups. Furthermore, we argue that, besides kinases, phosphatases should no longer be ignored in clinical trials, particularly in type II ECs, where the tumour suppressive phosphatase protein phosphatase type 2A (PP2A) is frequently mutated. Lastly, we discuss the therapeutic potential of targeting PP2A for (re)activation, possibly in combination with pharmacologic kinase inhibitors.

## 1. Introduction

Cancer is responsible for 1 in 6 deaths worldwide, and is thereby the second leading cause of mortality [1]. In 2012, there were on estimation 6.6 million new cancer cases and 3.5 million cancer deaths, specifically for women. Endometrial cancer accounted for 2.1% of these cancer deaths, and 5% of these cancer cases, making it the fourth most common cancer in women. Moreover, global incidence rates for endometrial cancer are still rising each year. In the USA for example, incidence rates have risen about 24% over a period of 23 years and are expected to rise another 35% by 2030 [2]. Additionally, even though technological advancements have improved standard therapies, mortality rates for endometrial cancer are still increasing. Over the last 15 years, there has been an increase in endometrial cancer related deaths of about 15% [1,2,3,4,5], supporting the notion that therapies for endometrial cancer need to be urgently revised and improved. Recent advances in the understanding of the molecular mechanisms of gynaecological cancers and the discovery of potential molecular markers through large-scale genomics studies have paved the way to implementing targeted therapeutics, an approach already successfully used in other cancers.

In this review, we discuss the therapeutic potential of targeting phosphorylation-dependent oncogenic signalling, particularly in type II endometrial cancers. The initial focus is on the use of pharmacologic kinase inhibitors as targeted therapeutics. However, the biochemical antagonists of protein kinases, the protein phosphatases, have more recently also come into the limelight as potential targets in cancer, particularly in type II endometrial carcinomas, where the tumour suppressive phosphatase protein phosphatase type 2A (PP2A) is frequently mutated. Moreover, PP2A (re)activation therapies may be of significant relevance to improve kinase inhibitor treatments. Therefore, we argue that targeted therapies, that take the tumour type and molecular profile of endometrial cancers into account, should be implemented on a more rational basis in clinical trials.

## 2. Endometrial Carcinomas: Histologic Classification and Diagnosis

Most endometrial cancers (97%) are epithelial lesions arising from the lining of the uterus, known as endometrial carcinomas (ECs) [2]. In 1983, Bokhman classified ECs into two types, based on the histopathology of the tumour [6]. Type I EC is composed of oestrogen-dependent, mostly low-grade endometrioid tumours and represents up to 80% of all ECs [6,7]. Endometrioid EC generally has a favourable prognosis with a five-year-survival rate of more than 80% due to its indolent clinical course and early stage detection [8]. On the other hand, type II ECs are high-grade by definition, with a poor prognosis, and mainly include three distinct histologies: Serous adenocarcinomas (10–20%), clear cell adenocarcinomas (<5%) and carcinosarcomas (<5%) [9,10,11,12,13,14,15,16]. Carcinosarcomas consist of a sarcoma as well as a carcinoma component. This carcinomatous component can display serous or endometrioid histology. However, most carcinosarcomas develop from a serous precursor and consequently have more similarities with high-grade serous tumours [17]. Hence, carcinosarcomas are treated like type II ECs [18]. Here, we will mainly focus on the serous ECs, which are most common within the type II ECs. Generally, type II ECs are oestrogen-independent and have an aggressive clinical course with late stage detection and a high tendency for early metastasis and extra-uterine spread. For example, several studies reported extra-uterine disease at the time of diagnosis in over 65% of patients with serous EC compared to only 4% of patients with type I endometrioid EC [9,19,20,21]. Serous EC was found to spread most often to the pelvic and paraaortic lymph nodes (in more than 40% of cases) but also to the cervix, ovaries and lungs [16,21,22,23]. Owing to these aggressive characteristics, serous EC has a high recurrence rate and a very poor five-year survival rate of less than 30% [9,12,19,24]. Consequently, serous ECs are responsible for almost half of the EC-related deaths, even though they represent only 10–20% of all ECs [9]. This indicates more effort has to be made in terms of treatment for patients with type II EC.

To date, standard treatment for all endometrial cancers is surgery, followed by adjuvant therapy based on grade and stage of the tumour [23]. Therefore, it is important to diagnose patients with the correct type of EC, in order to avoid over- or undertreatment. Several tools are used for diagnosing patients with endometrial cancer. Ideally, an accurate diagnosis is made without the need for surgery. In these pre-operative analyses, tools like the Papanicolaou (Pap) test can be used. However, the sensitivity of Pap tests in the detection of suspicious glandular cells is very low [25]. Additionally, transvaginal ultrasound (TVU), magnetic resonance imaging (MRI) and computed tomography (CT) scans can help with pre-operatively diagnosing tumour stage. However, the results from these tests are not always conclusive, resulting in incorrect clinical estimations in over 20% of cases [26,27,28]. Therefore, the golden standard technique for diagnosing endometrial cancer consists of two procedures. First, an endometrial biopsy tissue is taken, and histological analysis of this tissue is used to decide on tumour grade and subtype based on Bokhman’s classification. Second, surgical staging is performed to accurately determine the extent of the disease according to the staging system developed by the International Federation of Obstetrics and Gynaecology (FIGO) [23,29]. Determining tumour subtype solely based on histological information is, however, rather subjective and prone to error, resulting in poor reproducibility. This was demonstrated in a study by Gilks et al., in which 3 independent reviewers disagreed about the grade of the tumour (low-grade versus high-grade) in 36% of the cases [30]. Moreover, also serous versus endometrioid histology was found to be a frequent point of disagreement. This means that patients with high-grade serous EC could be diagnosed as low-grade endometrioid EC and consequently receive inadequate surgery and treatment. Hence, there is a clear need for a more objective classification of the tumours, which recently became feasible through analysis of the genomic profiles of endometrial cancers. For example, Ratner et al. proposed the use of miRNA signatures to differentiate between EC subtypes [31]. However, mutational analyses of cancers made an even more elaborate classification possible, based on the molecular gene profiles of the tumours.

## 3. Genomic Classification of Endometrial Carcinomas

Since the human genome project in 1990 and the emergence of next-generation sequencing, the focus of cancer research has shifted towards the molecular level [32]. This led to the identification and characterisation of many molecular alterations associated with different cancers. In turn, these molecular alterations helped us to understand the underlying mechanisms of tumorigenesis.

One of the pioneering initiatives investigating the molecular profile of several cancers was The Cancer Genome Atlas (TCGA). Initially, they analysed 3281 tumours from twelve cancer types, among which endometrial carcinoma [33]. TCGA objectively classified endometrial tumours based on their molecular profiles, revealing four major genomic groups: Polymerase ε (POLE) ultra-mutated, microsatellite instability (MSI), copy number low and copy number high [34]. The first three groups display endometrioid histology while the last group mostly involves serous histology. Based on this classification, Kommoss et al. designed the molecular classification tool ProMisE (Proactive Molecular Risk Classifier for Endometrial Cancer) [35]. This tool can be applied to endometrial biopsies and consistently identifies four prognostic genomic groups. ProMisE was successfully validated in a retrospective cohort and is now being clinically evaluated. Correct genomic classification of the tumour can help to form a more objective and accurate diagnosis of the tumour type, consequently leading to the right therapy. Nevertheless, high-grade tumours like serous ECs are always treated with adjuvant therapy (e.g., chemotherapy and/or radiation therapy), even though they show low response rates [36,37,38,39,40,41]. This emphasises the need for new predictive biomarkers and improved targeted therapies within this fourth EC subgroup, in particular for patients with resistant and recurrent type II ECs.

## 4. Molecular Markers in Endometrial Carcinomas

Molecular analysis of endometrial tumours allowed for the identification of mutations in oncogenes and tumour suppressor genes, thereby exposing the oncogenic signalling pathways for these cancers. ECs revealed to be most frequently mutated in *TP53*, *PPP2R1A*, *FBXW7*, *PIK3CA*, *PTEN*, *ARID1A*, *CTNNB1* and *KRAS*. Additionally, type II ECs also frequently have *HER2* gene amplifications. An overview of the frequency of these mutations in type I and II ECs can be found in Table 1.

*TP53* encodes the transcription factor and tumour suppressor p53, and is the most commonly mutated gene in human cancers [67]. However, *TP53* mutations occur at a much lower frequency in type I ECs (<15%) (Table 1). Remarkably, high-grade endometrioid ECs have more frequent mutations in *TP53* (up to 30%) [34]. This indicates *TP53* mutations are associated with a poor prognosis in endometrial cancer, which is also demonstrated by cBioportal survival data [56,57]. These survival data report a five-year overall survival rate of 60% for patients with *TP53* mutations compared to up to 90% for patients without *TP53* mutations. So far, therapeutic targeting of p53 has mostly been limited to pre-clinical studies testing small molecules, but toxicity towards healthy cells was a frequent problem [68].

The second most mutated gene in type II ECs turned out to be *PPP2R1A*, encoding the Aα subunit of the Ser/Thr-specific PP2A phosphatase, a known tumour suppressor [69]. PP2A phosphatases, for instance, regulate growth factor-induced Raf/Extracellular signal-Regulated Kinase (ERK) signalling, phosphatidylinositol-4,5-bisphosphate 3-Kinase (PI3K)/Akt signalling, mammalian target of rapamycin (mTOR) signalling, and WNT signalling [70,71,72]. Remarkably, somatic mutations in *PPP2R1A* occur at high frequencies in type II ECs (up to 40%), while only a low percentage is found in type I endometrioid ECs (<7%) (Table 1). Additionally, the few *PPP2R1A* mutations found in endometrioid ECs are mostly correlated with high-grade endometrioid EC, suggesting *PPP2R1A* mutations are associated with aggressiveness of the tumour and poor patient outcome [73]. Moreover, cBioportal survival data indicate a five-year survival rate of 50% for patients with serous EC harbouring *PPP2R1A* mutations compared to 80% for patients without *PPP2R1A* mutations [56,57]. However, these data only include 12 patients. Therefore, a larger group of patients with type II ECs will need to be investigated in order to get more conclusive results about the prognostic marker potential of *PPP2R1A*. Interestingly, *PPP2R1A* mutations occur early during progression in the precursor lesions and are able to distinguish serous EC from the clinicopathological similar ovarian high-grade serous carcinomas, which rarely harbour *PPP2R1A* mutations [44,52].

*FBXW7* encodes the tumour suppressive FBOX protein, a component of the Skp, Cullin, F-box (SCF)-ubiquitin ligase complex [74]. This complex targets phosphoprotein substrates for ubiquitination and subsequent proteasomal degradation. *FBXW7* mutations are most frequently reported in type II ECs (Table 1) and mainly affect the substrate binding WD repeats of the FBOX protein resulting in loss of function of the SCF-complex and hence (onco)protein accumulation. Interestingly, mTOR is one of the substrates of this SCF-complex. Consequently, inactivating mutations in *FBXW7* can result in PI3K pathway activation through mTOR stabilisation [75].

The PI3K pathway in type II ECs is also often affected by recurrent mutations in *PIK3CA* and *PTEN* (Table 1). *PIK3CA* encodes the p110α catalytic subunit of the class IA PI3Ks, which catalyse phosphorylation of phosphatidylinositol 4,5-bisphosphate (PIP_2_) resulting in phosphatidylinositol 3,4,5-trisphosphate (PIP_3_). Thus, *PIK3CA* mutations lead to the constitutive activation of PI3K signalling [76]. *PTEN* encodes the phosphatase and tensin homolog (PTEN), a lipid as well as a protein phosphatase. As a lipid phosphatase, PTEN is the functional antagonist of PI3K, and specifically dephosphorylates PIP_3_. Hence, inactivating mutations in *PTEN* mostly result in overactivation of PI3K signalling. *PTEN* is mutated at low frequencies in type II ECs while mutated at very high frequencies (up to 84%) in type I endometrioid ECs (Table 1). The higher frequency of *PTEN* mutations reported in type II carcinosarcomas compared to the other type II ECs could be explained by its biphasic nature, containing carcinoma and sarcoma elements. Specifically, *PTEN* mutations were reported in the carcinoma component resembling endometrioid histology and not in the component resembling serous histology [77]. However, here we made no distinction between the mutational profiles of the serous-like and endometrioid-like carcinomatous component within the carcinosarcomas. Nevertheless, most carcinosarcomas resemble type II serous tumours. This is also indicated by their general mutational profile, which is more closely related to type II serous ECs than to type I endometrioid ECs (Table 1) [17,78].

*ARID1A* encodes the BAF250A tumour suppressor and is functionally involved in the SWI/SNF chromatin-remodelling complex [79]. *ARID1A* mutations are less common in type II ECs than in type I ECs (Table 1). Interestingly, *ARID1A* mutations can result in PI3K pathway activation via downregulation of PI3K interacting protein 1 (PIK3IP1). Furthermore, inhibition of the EZ2H methylesterase in *ARID1A* mutated ovarian cancer cells results in synthetic lethality, suggesting EZ2H as a potential new therapeutic target for *ARID1A* mutated tumours [80,81,82,83].

*CTNNB1* encodes β-catenin and is mutated at lower frequencies in type II ECs compared to type I ECs (Table 1). β-catenin is an important component of the canonical WNT signalling pathway and stimulates transcription of genes associated with proliferation and cell survival [84]. β-catenin is negatively regulated by glycogen synthase kinase 3β (GSK-3β) which phosphorylates β-catenin leading to its degradation. Most *CTNNB1* mutations result in evasion of GSK-3β-mediated phosphorylation resulting in nuclear accumulation and consequently, increased cell proliferation [85].

Furthermore, *KRAS* mutations have been reported in ECs, although at low frequencies for both EC types (Table 1). *KRAS* encodes the small GTPase K-Ras, which is an oncoprotein involved in the activation of several signalling pathways, including the PI3K and mitogen-activated protein kinase (MAPK) pathways, and in the activation of other small GTPases, such as RalA [86]. *KRAS* mutations result in Ras proteins with constitutively bound GTP, consequently activating these downstream oncogenic pathways.

Lastly, also *HER2* gene amplification was identified as a common genetic alteration in ECs, with a higher frequency in type II ECs compared to type I ECs (Table 1). *HER2* encodes the human epidermal growth factor receptor 2 (HER2), which belongs to the Epidermal Growth Factor Receptor (EGFR) family of Receptor Tyrosine Kinases (RTKs) and stimulates signal transduction via the PI3K and MAPK pathways [87]. Consequently, *HER2* amplification/overexpression can lead to oncogenic overactivation of the PI3K and MAPK pathways. Furthermore, *HER2* amplification/overexpression was found to be correlated with a worse prognosis for patients with EC [88].

In summary, the identification of these nine most altered genes and related signalling pathways involved in tumorigenesis of type II ECs, opens opportunities for the rational development of targeted therapies in the clinical management of these tumours. Remarkably, seven out of nine (*PPP2R1A*, *FBXW7*, *PIK3CA*, *PTEN*, *ARID1A*, *KRAS*, *HER2*) of the type II EC-associated genes encode proteins involved in regulation of the PI3K pathway. Furthermore, some of these genetic alterations (*PPP2R1A*, *KRAS* and *HER2*) can also lead to activation of the MAPK pathway. Thus, the logical next step would be to test therapeutics targeting the affected PI3K and/or MAPK pathways in patients with type II ECs. Specifically, based on the specific nature of the most recurrent molecular alterations found in type II ECs, the therapeutic targeting of phosphorylation-dependent oncogenic signalling seems a particularly promising strategy to improve current treatments—especially, since a large variety of pharmacologic kinase inhibitors, targeting activated PI3K or MAPK signalling, have already been developed. In addition, targeting of the counteracting protein phosphatases has recently moved into the limelight as novel promising cancer therapeutics, and may also be applied to specific molecular subsets of type II ECs.

## 5. Therapeutic Potential of Targeting Kinases and Phosphatases in Endometrial Carcinomas

### 5.1. Successes of Kinase Inhibitors as Targeted Cancer Therapies

During the last three decades, protein kinases have gained enormous interest as potential therapeutic targets in many common cancer types. A well-known example is the use of the US Food and Drug Administration (FDA)-approved Tyrosine Kinase Inhibitor (TKI) imatinib in treatment of patients with chronic myeloid leukaemia (CML) [89]. More than 90% of these patients are characterised by the presence of the Philadelphia chromosome and its oncogenic product, the constitutively active BCR-ABL tyrosine kinase. Imatinib inhibits this kinase and significantly improves outcome of patients with Philadelphia chromosome-positive CML. Another example can be found in treatment of non-small cell lung cancer (NSCLC), where two main targeted therapies affecting protein kinases are currently clinically applied. The first ones are the FDA approved EGFR tyrosine kinase inhibitors (e.g., erlotinib and gefitinib), which show high response rates in patients with NSCLC harbouring *EGFR* mutations [90,91]. The second ones are the FDA approved anaplastic lymphoma kinase (ALK) inhibitors (e.g., crizotinib and ceritinib), which show positive effects in tumours harbouring *ALK* gene rearrangements [92,93]. Lastly, also breast cancer therapy has successfully implemented targeted therapies in a molecularly stratified group. For example, patients with breast cancer tumours overexpressing the HER2 receptor (in up to 30% of cases) showed favourable response to the FDA-approved anti-HER2 monoclonal antibody Trastuzumab [94,95,96]. A list of other FDA-approved drugs targeting cancers with specific mutations can be found on the online tool OncoKB [97]. Therefore, from these examples, the overall image has emerged that by stratifying patients based on the affected gene/pathway, targeted therapies can prove to be much more effective. However, despite these positive results, this strategy is still not applied for treatment of endometrial cancers.

### 5.2. Targeted Therapies in Endometrial Carcinomas: Mainly Geared towards PI3K Signalling

**Figure 1:**
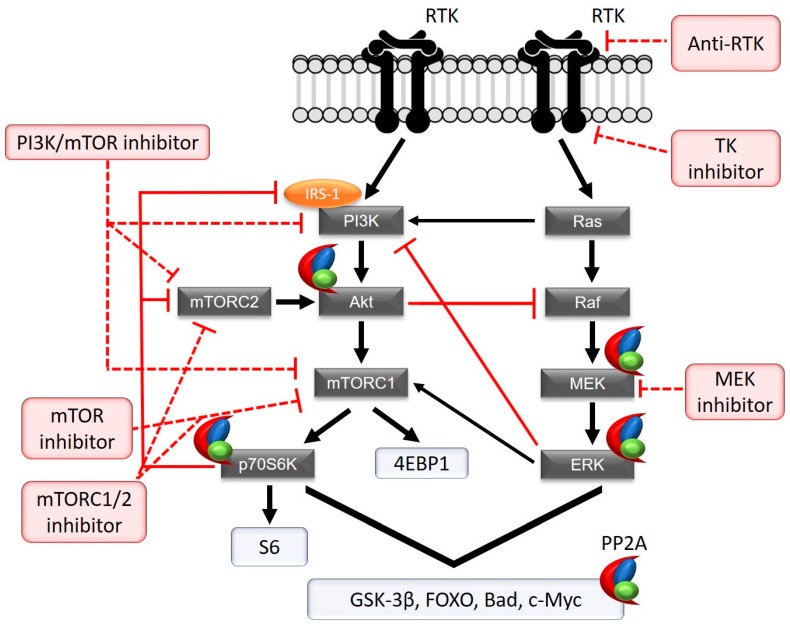
Schematic overview of the PI3K and MAPK pathways and important substrates. Black arrows represent activation. Red lines represent endogenous inhibition through feedback/cross-talk. Dotted red lines represent inhibition with pharmacologic kinase inhibitors. The heterotrimeric PP2A complex is represented in red (A subunit), blue (B subunit) and green (C subunit). Both pathways can be targeted at several levels using pharmacologic kinase inhibitors. However, single agent inhibitors targeting mTORC1 (e.g., everolimus) will deactivate the negative feedback loops from p70S6K to mTORC2 and PI3K. The use of a dual PI3K/mTOR inhibitor could circumvent this problem. There is also cross-talk between the PI3K and MAPK pathway, which could be evaded by using the combination of a PI3K and MAPK (e.g., MEK inhibitor) pathway inhibitor. Anti-RTKs (e.g., Trastuzumab) target the extracellular domain of the RTKs. TK inhibitors (e.g., Lapatinib) target the intracellular tyrosine kinase activity of the RTKs. Furthermore, PP2A acts as a tumour suppressor on many components of both pathways and should therefore be considered when targeting kinases. Additionally, it is an attractive target for activation, and hence PI3K and MAPK pathway downregulation, potentially in combination with kinase inhibitors. The PI3K and MAPK pathways have several substrates in common (GSK-3β, FOXO, Bad and c-Myc), which are involved in cell proliferation and cell survival. Some substrates, like FOXO and GSK-3β, are activated by Akt, independently of mTORC1. 4EBP1: eukaryotic translation initiation factor 4E-binding protein 1, Bad: Bcl-2-associated death promoter, FOXO: forkhead box protein, GSK-3β: glycogen synthase kinase 3β, IRS-1: insulin receptor substrate 1, mTOR: mammalian target of rapamycin, PI3K: phosphatidyl-4,5-bisphosphate 3-kinase, PP2A: protein phosphatase 2A, RTK: receptor tyrosine kinase, S6: ribosomal protein S6, TK: tyrosine kinase, MAPK: mitogen-activated protein kinase; ERK: extracellular signal-regulated kinase; MEK: mitogen-activated protein kinase kinase.

Therapies for endometrial cancer still tremendously lag behind. So far, the only FDA-approved targeted therapies for ECs are hormonal intervention (for hormone-dependent endometrioid ECs) and the immune checkpoint inhibitor pembrolizumab [98]. However, for type II ECs, there are no approved targeted therapies, even though these patients need it the most. Molecular analysis revealed the PI3K pathway to be most frequently involved in tumorigenesis of ECs (Table 1) [99]. This led to the clinical evaluation of targeted therapies against this pathway, which can be affected at different levels via multiple kinase inhibitors (Figure 1) [100]. Initially, clinical trials mainly tested derivatives from rapamycin (e.g., everolimus, temsirolimus), single mTOR kinase inhibitors primarily targeting the mTORC1 complex, in unstratified groups. However, the outcome with these single agent inhibitors turned out to be rather disappointing as reviewed by several research groups [101,102,103,104].

Two main biochemical mechanisms inherent to the PI3K pathway could explain for these disappointing results. The first mechanism is the presence, as in many signalling pathways, of negative feedback loops preventing overactivation of the PI3K pathway under normal conditions (Figure 1). Briefly, stimulation of the PI3K pathway leads to mTORC1 activation and, consequently, to phosphorylation and activation of its downstream substrate p70S6 kinase. In turn, p70S6 kinase inhibits PI3K signalling via two feedback loops [105]. The first one acts via phosphorylation and inhibition of the insulin receptor substrate (IRS)-1, a docking protein for PI3K. The second one acts via phosphorylation of mTORC2, leading to its inhibition. mTORC2 is located upstream of mTORC1 and is involved in Akt phosphorylation and activation. Pharmacologic mTORC1 inhibitors disrupt these negative feedback loops, resulting in constitutive activation of the PI3K pathway, thereby abolishing the effect of mTOR inhibition [106]. Furthermore, mTOR independent targets of Akt (e.g., Forkhead box (FOXO), GSK-3β) can also contribute to increased cell proliferation and survival [107].

Therefore, additional blocking of the PI3K pathway via dual mTORC1/2 inhibitors or PI3K/mTOR inhibitors was thought to be able to circumvent these problems associated with single mTORC1 inhibition. Indeed, pre-clinical studies testing mTORC1/2 and PI3K/mTOR inhibitors demonstrated improved anti-tumour activity compared to single agent mTOR inhibitors, although Shoji et al. reported no persistent effect of the PI3K/mTOR inhibitor in vitro and in vivo [108,109]. Furthermore, a phase II clinical trial where a dual PI3K/mTOR inhibitor was used had limited success due to its poor tolerability [110]. Nevertheless, patients with confirmed response all had mutations in the PI3K pathway. Additionally, Weigelt et al. showed endometrioid EC cell lines with mutations in the PI3K pathway to be more sensitive to PI3K and mTOR inhibitors [111], while *KRAS* mutant ECs did not react to mTORC1 treatment in a phase II clinical trial [112]. This all suggests that PI3K/mTOR pathway inhibitors are more efficient in tumours with activated PI3K signalling.

This view was further sustained by several pre-clinical trials in which serous EC cell lines with and without *HER2* amplifications were used, and three PI3K pathway inhibitors were tested (mTORC1/2, PI3K and a dual PI3K/mTOR inhibitor) [113,114,115]. In all of these studies, the *HER2* amplified cells (with activated PI3K pathway) were more sensitive towards the PI3K pathway inhibitors than the wildtype *HER2* cell lines. Furthermore, some of these cell lines had additional mutations in *PIK3CA*, which rendered them even more sensitive in case of dual PI3K-mTORC1/2 inhibition [113]. This certainly advocates for the implementation of genotype-dependent patient stratification in clinical trials, so that kinase inhibitors could be tested on a more rational basis. Nevertheless, in two phase II studies of mTORC1 inhibitors, *PIK3CA* and *PTEN* mutations in EC tumours could not predict patient outcome [112,116], but this could be due to the use of an agent targeting just a single kinase (as explained above). Until now, however, most clinical trials for endometrial cancers had no biomarker restrictions [101], likely also explaining the poor results.

The second mechanism, likely to be responsible for the poor response rates to mTOR inhibitors involves the phenomenon of “cross-talk”, in which there is (altered) signalling to other pathways—here, most often, the MAPK pathway. Cross-talk between the PI3K and MAPK pathways is well-established and elaborately reviewed by Mendoza et al. [107]. Briefly, both PI3K and MAPK pathways can regulate each other positively as well as negatively (Figure 1). For example, Akt (PI3K pathway) phosphorylates and inhibits Raf (MAPK pathway), thereby blocking MAPK signalling. On the other hand, ERK (MAPK pathway) negatively regulates PI3K and stimulates mTORC1. Therefore, in case of PI3K pathway inhibition, cross-inhibition from Akt to Raf is relieved, resulting in activation of the MAPK pathway and hence, treatment resistance. Furthermore, since the PI3K and MAPK pathways often target the same substrates (e.g., FOXO, c-Myc, Bad, GSK-3β), cross-activation will ensure these substrates remain activated even after PI3K pathway inhibition. Such cross-activation due to PI3K pathway inhibition is also reported in other cancers. For example, inhibition of the PI3K pathway resulted in MAPK pathway activation in NSCLC cell lines [117]. Additionally, also breast cancer studies reported this inhibitor-induced activation of the MAPK pathway after administration of a single mTOR inhibitor [118]. Furthermore, the authors of this study observed that a Mitogen-activated protein kinase kinase (MEK) inhibitor was able to reduce MAPK activation and reduce cell growth. Remarkably, the combination of the mTOR and MEK inhibitor had an additive effect, resulting in stronger cell growth reduction. Furthermore, in EC cell lines, PI3K/mTOR inhibition resulted in MAPK pathway activation [109], and the combination of a MEK inhibitor and PI3K/mTOR inhibitor improved anti-proliferative effects.

Furthermore, pharmacologic targeting of HER2 has also gained interest as a potential therapeutic strategy for patients with type II ECs, especially since positive results were obtained for patients with *HER2* positive breast cancer as mentioned in Section 5.1. Therefore, anti-HER2 compounds like Trastuzumab (anti-HER2 antibody) and Lapatinib (small molecule TKI) were also tested in ECs. However, single agent targeting of HER2 with Trastuzumab or Lapatinib had no effect in vitro (serous EC cell lines) and in vivo (xenografts) [119]. In contrast, dual inhibition with Trastuzumab and Lapatinib in serous EC xenografts showed significant anti-tumour activity and resulted in decreased phosphorylation of downstream PI3K and MAPK signalling proteins. Remarkably, this effect was only observed in the *HER2*-amplified serous EC cell lines, indicating the importance of patient stratification based on *HER2* status of the tumour. This important paradigm was further underscored in several publications of Santin et al., in which the responses of serous EC cell lines with or without *HER2* amplification to HER2 inhibitors were assessed [120,121,122,123,124]. They consistently observed that the *HER2* amplified cell lines showed a higher sensitivity for these targeted inhibitors than the cell lines without *HER2* amplification. Furthermore, *HER2* amplified cells with additional *PIK3CA* mutations showed resistance to the anti-HER2 monoclonal antibody Trastuzumab, while *HER2* amplified cells with wildtype *PIK3CA* were sensitive towards Trastuzumab [120]. This clearly indicates the benefit of molecular stratification and advocates the implementation of such stratification in clinical trials. Not surprisingly, clinical trials testing the efficacy of Trastuzumab and Lapatinib in unstratified patient populations had poor response rates [125,126]. Furthermore, these trials tested single agent inhibitors of the PI3K pathway, probably activating several resistance mechanisms (as mentioned above).

Ongoing or recently completed clinical trials for type II serous ECs registered in ClinicalTrials.gov were searched using the keyword “serous endometrial adenocarcinoma”. Strikingly, out of 36 matching clinical trials, only 16 focussed on targeted therapies with or without combination with adjuvant therapy. The other 20 trials solely used adjuvant therapies. Remarkably, only three out of 16 trials using targeted therapies (NCT02491099, NCT00506779, NCT01367002) specifically recruited type II serous ECs and had biomarker restrictions. Currently, two of these clinical trials (NCT02491099 and NCT01367002) are ongoing and promising results in favour of molecular stratification have already been obtained in NCT01367002 [127]. The other 13 trials testing targeted therapies, unfortunately, included both type I and type II ECs in their study and did not select patients based on their mutational profiles. Once more, this clearly illustrates that, even up to date, with molecular data of type II ECs at hand, and despite the proven clinical successes of targeted therapies in molecularly well-defined tumours, a change in mind-set among clinical oncologists is definitely still needed to improve the setup, and thereby (hopefully) the outcome, of novel clinical trials in (type II) EC.

Summarised, several (pre-)clinical results have indicated that dual pathway inhibition could evade the problem of cross-talk and improve treatment efficacy in PI3K or MAPK pathway driven endometrial cancers. Furthermore, these studies have illustrated that molecular stratification of ECs is critical in efficient testing of kinase inhibitor therapies. On the other hand, it is also known that the clinical success of kinase inhibitors in cancer therapy is often of limited duration, as over time, many patients develop therapy resistance, for example by acquiring mutations in the drug target [128]. However, based on the biochemical logic that not just a kinase, but both kinases and phosphatases regulate the phosphorylation of any phosphoprotein under normal conditions, there is an emerging view that inhibition of phosphorylation-dependent oncogenic signalling may only be efficiently achieved by targeting both kinases and phosphatases [129,130]. Specifically, one cannot expect to efficiently revert hyperphosphorylation of an oncoprotein just by inhibiting the oncogenic kinase involved, if the counteracting tumour suppressive phosphatase is no longer active. In terms of avoiding acquired kinase inhibitor resistance, a combination therapy of a kinase inhibitor and a phosphatase activator may indeed be much more effective [130]. Particularly for type II ECs, where *PPP2R1A* is mutated at high frequencies, opportunities may be ahead to exploit the phosphatase PP2A for therapeutic purposes.

### 5.3. Targeting the Phosphatase PP2A in Type II Endometrial Carcinomas

**Figure 2:**
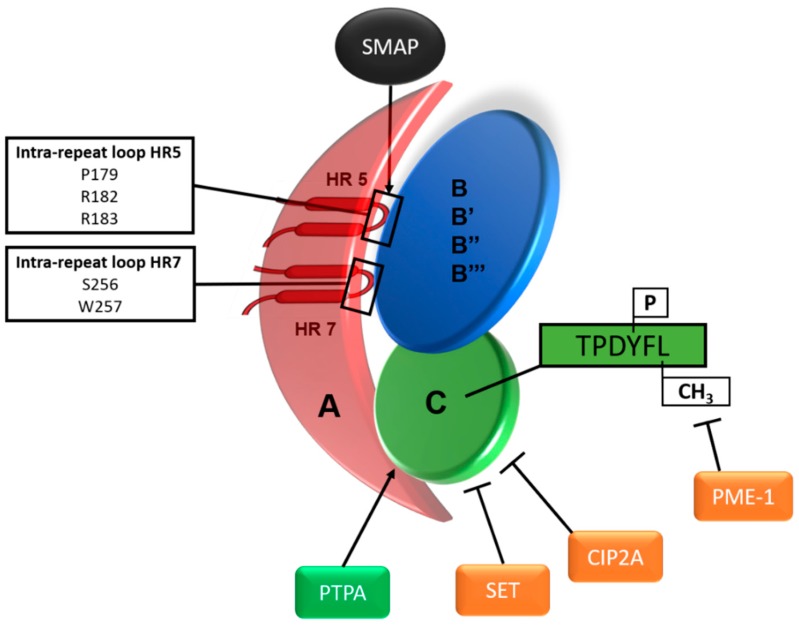
The heterotrimeric PP2A complex with activating and inactivating mechanisms. Heat repeat (HR) 5 and 7 are represented with their intra-repeat loops in subunit A. These intra-repeat loops harbour the most recurrent *PPP2R1A* hotspot mutations identified in type II ECs. Endogenous inhibition of PP2A can occur via SET, CIP2A and PME-1, which act on the C subunit. TPDYFL is the conserved motif in the C-terminal tail of the catalytic C subunit. Phosphorylation of the tyrosine (Y) is thought to cause inactivation, while methylation of the carboxyterminal leucine (L) of the C subunit promotes binding of specific B subunits, and thereby assembly of active trimers. PME-1 demethylates this leucine, and stabilises inactive PP2A complexes. PTPA is necessary for endogenous activation of inactive PP2A complexes. SMAP is a small molecule activator of PP2A, which binds at heat repeat 5 in close proximity to the hotspot mutations in *PPP2R1A*. The vast array of regulatory B subunits allows for the targeting of PP2A to many different substrates. A: Scaffolding A subunit; B, B′, B″, B′′′: The four families of regulatory B subunits, each containing several isoforms; C: catalytic C subunit; CH_3_: methyl group; CIP2A: cancerous inhibitor of PP2A; HR: heat repeat; P: phosphate group (PO_4_^3−^); PME-1: PP2A methylesterase 1; PTPA: phosphatase 2A phosphatase activator; SET: Suvar/Enhancer of zeste/Trithorax; SMAP: small molecule activator of PP2A.

The tumour suppressive nature of PP2A was first established through the observation that the tumour promotor okadaic acid (OA), which specifically inhibits PP2A, resulted in cellular transformation in mouse skin [69]. Later on, functional studies in several human epithelial cells have underscored that inhibition of PP2A (typically achieved by expression of SV40 small T antigen) is an absolute requirement to fully transform an immortalised human cell, despite the overt activation of oncogenic kinases (e.g., typically downstream of an established oncogene, such as *RAS*) [131]. PP2A phosphatases are heterotrimers composed of a catalytic C subunit, a scaffolding A subunit, and a regulatory B subunit (Figure 2) [132]. SV40 small T antigen was shown to inhibit PP2A through displacement of the PP2A B subunits [69]. The A and C subunit each have two isoforms, α and β, of which the α isoform is the most common. The A subunit forms a scaffold for the catalytic C and regulatory B subunits and is composed of 15 HEAT repeats, each consisting of two anti-parallel α-helices connected by an intra-repeat loop (Figure 2) [132]. These intra-repeat loops allow protein-protein interactions with the catalytic C subunit and regulatory B subunits. More precisely, the B subunits are known to bind to HEAT repeats 1–10 while the C subunit binds to HEAT repeats 11–15. [133]. The regulatory B subunits are divided into four families (B/PR55/B55, B′/PR61/B56, B″/PR72, B′′′/Striatins), each containing several isoforms, and thereby resulting in a vast array of PP2A holoenzymes [132]. The B subunits are responsible for subcellular localisation and determine substrate specificity of the PP2A complexes. This way, PP2A complexes negatively regulate a variety of signalling pathways involved in carcinogenesis (Figure 1) [71,72,134]. Consequently, inactivation of PP2A has been associated with several human cancers [135,136,137], and increased rates of spontaneous or carcinogen-induced oncogenesis in mice [138,139,140,141]. Therefore, therapeutic targeting of PP2A has gained interest and has been focussing on the direct or indirect (re)activation of PP2A (reviewed in [142]). How exactly this should be achieved, might in part be determined by the mechanism(s), if any, by which PP2A is inactivated in the tumour.

Strikingly, most type II EC-associated heterozygous missense mutations in *PPP2R1A*, encoding the Aα subunit isoform of PP2A, cluster in HEAT repeats 5 and 7, encoded by exon 5 and 6, respectively. These *PPP2R1A* mutations frequently recurred at the same positions (P179, R183 and S256) across different cancer types, as indicated by several comprehensive studies [143,144,145]. In type II ECs, these so-called *PPP2R1A* hotspot mutations were recurrently found in codons: P179, R182, R183 (HEAT repeat 5) and S256, W257 (HEAT repeat 7) (Figure 2) [17,44,45,48,52,54,144,146,147]. Remarkably, based on structural studies, the same residues or neighbouring residues were predicted to be important for interaction with the B subunits [133,148]. However, experimental evidence for this hypothesis revealed a much more sophisticated image, in that mutations at these positions indeed resulted in binding deficiencies of Aα mutants with several regulatory B subunit types, but specifically preserved binding to others, most notably to B56δ and B56γ [149,150]. Some mutations, e.g., p.(P179R), also diminished binding to the catalytic C subunit [149]. Moreover, Haesen et al. suggested a dominant negative mechanism for these mutants, as the PP2A trimers that could still be formed proved catalytically impaired through the increased recruitment of a cellular PP2A inhibitor, TIPRL1 [149]. Accordingly, ectopic expression of the Aα mutants increased anchorage-independent cell growth in vitro, xenografted tumour growth in vivo, and resulted in hyperactivation of, again, the PI3K/Akt/mTOR pathway. Interestingly, downregulation of the MAPK pathway was seen in these conditions, suggesting cross-activation might not be an issue. Thus, type II ECs with *PPP2R1A* driver mutations might be sensitive towards single pathway (PI3K/mTOR) inhibitors. Hence, these results open a window of opportunity for the use of kinase inhibitors targeting the PI3K/mTOR pathway in type II ECs, harbouring an oncogenic mutation in *PPP2R1A*, potentially in combination with pharmacologic PP2A activators.

#### 5.3.1. Direct Targeting of PP2A

Recently, a lot of interest has gone into the discovery of Small Molecule Activators of PP2A (so-called SMAPs) that appear to be able to allosterically activate PP2A. In 2014, the FDA-approved tricyclic neuroleptic drug perphenazine was found to have anti-proliferative effects in T-cell acute lymphoblastic leukaemia (T-ALL) through binding and activation of PP2A, and subsequent downregulation of both the PI3K and MAPK pathways [151]. At first, the extrapyramidal side effects and high concentrations necessary for PP2A activation made the perphenazines not appealing as anticancer drugs. However, Kastrinsky et al. reengineered these drugs to abrogate the CNS activity and enhance the anti-proliferative effects, resulting in the new molecular class of “small molecule activators of PP2A” (SMAPs) [152]. SMAPs were able to increase PP2A activity in *KRAS* mutant lung cancer cells, resulting in significantly decreased cell survival [153]. This was further established in lung cancer cell xenografts in mice, where SMAP treatment significantly inhibited tumour growth. Moreover, single agent SMAP treatment was as efficient as the combination treatment of an Akt (PI3K pathway) and MEK (MAPK pathway) inhibitor. This indicates SMAPs are able to inhibit both PI3K and MAPK pathway simultaneously, hence eliminating the problem of cross-activation of oncogenic pathways when using a single kinase (pathway) inhibitor, as well as avoiding potential tolerance problems of combinations of more than one kinase inhibitor. Furthermore, castration-resistant prostate cancer cells showed sensitivity towards SMAP treatment in vitro, as well as in xenografts [154]. The exact mechanism by which SMAPs are able to activate PP2A is not known yet, although Sangodkar et al. convincingly reported direct binding of SMAPs to the Aα subunit of PP2A (Figure 2). More precisely, residues K194 and L198 within HEAT-repeat 5 were necessary for the interaction with these compounds [153].

These pre-clinical data indicate it might be interesting to test SMAPs as treatment for ECs, which are mainly MAPK and PI3K pathway driven. Moreover, SMAPs might be able to re-activate PP2A in type II ECs which have frequent *PPP2R1A* mutations. However, SMAPs bind in close proximity (K194, L198) to the residues of the Aα subunit that are most frequently mutated (P179, R182, R183) in type II ECs [153]. Therefore, these mutations might influence SMAP binding to the Aα subunit and result in resistance to SMAPs. Future research into this potential issue should eventually allow to clarify whether *PPP2R1A* could be used as a predictive marker for SMAP treatment in type II ECs.

#### 5.3.2. Indirect Targeting of PP2A

Besides *PPP2R1A* mutations, other mechanisms of PP2A dysfunction in endometrial cancers have been described (Figure 2). Very commonly, PP2A inactivation in cancer occurs through overexpression of the endogenous PP2A inhibitors SET (Suvar/Enhancer of zeste/Trithorax) and CIP2A (cancerous inhibitor of PP2A), for example in chronic myeloid leukaemia and in many solid tumour types [136]. Therefore, many therapeutic strategies to target PP2A focus on the inhibition of these inhibitors, thereby indirectly re-activating PP2A. For example, FTY720 is a compound that is able to inhibit SET, resulting in increased PP2A activity [142]. In endometrial cancer, CIP2A overexpression is observed in endometrioid ECs, and siRNA mediated knockdown resulted in a decreased oncogenic phenotype [155]. However, to our knowledge no studies have investigated CIP2A or SET overexpression in type II ECs yet. cBioportal data reveal *CIP2A* gene amplification in 4.65% of serous ECs, albeit in a total of just 43 cases [56]. Therefore, involvement of CIP2A or SET in carcinogenesis of type II ECs needs to be further explored, potentially leading to more therapeutic options for this disease.

PP2A inactivation can also occur through aberrant post-translational modifications of the C-terminal tail of the catalytic C subunit (Figure 2). These modifications occur on the conserved C-terminal motif (TPDYFL), where phosphorylation of the tyrosine (Y) results in PP2A inactivation, and methylation of the carboxyterminal leucine (L) is required for binding select B-type subunits and assembly of active PP2A trimers [156]. Consequently, components inhibiting phosphorylation or promoting methylation generally lead to activation of PP2A.

PP2A demethylation is catalysed by the PP2A methylesterase PME-1, and increased PME-1 expression has been associated with tumour progression in human malignant gliomas [157]. Notably, PME-1 overexpression was also observed in 83% (24/29) of tumours from patients with type I endometrioid ECs [158], and PME-1 overexpression in endometrioid EC cell lines resulted in increased cell proliferation and anchorage-independent growth. Xenograft experiments confirmed these in vitro data, showing increased tumour growth in case of PME-1 overexpression. PME-1 overexpression was associated with a strong reduction in PP2A activity, consequently leading to increased ERK and Akt phosphorylation. As expected, PME-1 knockdown increased PP2A activity leading to decreased phosphorylation and hence, decreased activation of ERK and Akt. This indicates that targeting PME-1 could be feasible for re-activation of PP2A. Furthermore, addition of an Akt inhibitor further decreased Akt phosphorylation, suggesting combination therapy of a PME-1 and an Akt inhibitor could enhance anti-tumour activity even more [158]. Recently, another study confirmed the potential of PME-1 inhibition as an anti-cancer treatment in EC. They reported decreased tumour growth in xenografts after PME-1 depletion using shRNA [159]. Furthermore, they tested two pharmacologic inhibitors of PME-1, ABL-127 and AMZ-30, in type I EC cell lines. ABL-127 was the most potent inhibitor, increasing PP2A activity by 45%. However, pilot studies testing ABL-127 did not reduce tumour growth. Altogether, these date indicate the potential of PME-1 inhibition as treatment for type I ECs, while not tested for type II ECs. cBioportal reports *PPME1* (encoding PME-1) amplification in 2.33% of type II serous ECs on a total of 43 cases [56]. Despite apparent low frequencies, it is definitely worthwhile to further explore PME-1 inhibition in type II ECs, which could lead to new therapies for this disease.

Another important recurrent mechanism for genomic PP2A inactivation found in several human cancers, involves haploinsufficiency of *PPP2R4*, caused by heterozygous loss or mono-allelic loss-of-function mutations. Heterozygous loss of *PPP2R4* was found in about 20% of all endometrial cancers with up to 70% for specifically the type II endometrial carcinosarcomas [56,57,139]. *PPP2R4* encodes the phosphatase 2A phosphatase activator (PTPA), an essential cellular PP2A activator, necessary for the generation of active PP2A complexes. Sents et al. demonstrated loss-of function in five cancer-associated PTPA mutants [139]. These mutants had decreased PP2A-C binding and a reduced ability to reactivate PP2A in vitro. Furthermore, ectopic expression of these PTPA mutants in PTPA depleted HEK-TER cells could not rescue the oncogenic phenotype in vitro (anchorage-independent growth) and in vivo (xenograft growth), while ectopic expression of WT PTPA could. Moreover, PTPA-deficient mice had significantly impaired PP2A activity, decreased methylation, and spontaneously developed tumours [139]. These data further indicate PP2A could be a new therapeutic target, in this case especially for type II endometrial carcinosarcomas, where heterozygous loss of *PPP2R4* occurs in up to 70% of cases. In particular, it would be of interest to investigate whether SMAPs might be able to activate the inactive PP2A complexes thereby compensating for the lack of functional PTPA.

## 6. Conclusions and Future Perspectives

Molecular alterations in type II ECs hold major potential for the development of targeted therapies which, according to the most recurrent alterations, should be focused on the inhibition of phosphorylation-dependent oncogenic signalling in the PI3K and MAPK pathways. To date, there are still too few clinical trials specifically for type II ECs. Additionally, most of these trials mainly focus on kinase inhibitors without stratifying patients based on the affected pathway, even though this is successfully done in other cancers. Consequently, clinical trial results for ECs have been rather disappointing. Furthermore, there is emerging evidence that the enzymes counteracting the protein kinases, the protein phosphatases, also need to be considered in order to efficiently inhibit these oncogenic pathways. This seems particularly important for type II ECs, which harbour frequent mutations in the tumour suppressive phosphatase PP2A.

In order to improve clinical trials, we propose the implementation of a dual stratification system based on the molecular profile of the tumours. Firstly, patients need to be stratified based on tumour type, in which tools like ProMisE could help to make a more objective stratification possible. To date, most clinical trials do not distinguish between endometrial cancer types. This is particularly disadvantageous for type II ECs, since they account for only 10–20% of ECs. Accordingly, results are biased towards the more common type I ECs and no conclusive results can be obtained for patients with type II EC even though they need targeted therapies the most. Since type II ECs are less common, the number of available patients may be a problem. Therefore, the implementation of multi-institutional clinical trials should be encouraged. Secondly, patients need to be stratified based on their molecular alterations and, hence, affected pathways. Accordingly, kinase inhibitors can be tested on a more rational basis, targeting the pathway that indeed shows oncogenic alterations. Furthermore, it will also be important to take the status of the tumour suppressive phosphatase PP2A into account, whose inactivation in fact also results in upregulation of the PI3K/Akt/mTOR pathway. Additionally, direct or indirect targeting of PP2A for (re)activation could be a potential new therapy for type II ECs. In particular, the new molecular class of PP2A activators, SMAPs, seem promising to achieve direct activation of PP2A, and consequently, suppression of oncogenic signalling. The combination of both a kinase inhibitor and a phosphatase activator is emerging as the most promising novel therapeutic approach, which could help to circumvent problems like pathway cross-talk and acquired kinase resistance, thereby improving treatment efficacy.

Therefore, therapeutic approaches for type II ECs, so far, have benefited too little from the available molecular data, but it is definitely not too late to improve future clinical trials in order to obtain effective targeted therapies for type II ECs.

## Figures and Tables

**Table 1:** Most common genetic alterations in type I and type II endometrial carcinomas (EC). Percentages in the header refer to all EC cases; percentages in the table refer to each EC subtype.

Common Genetic Alterations	Type II Serous Carcinoma (10–20%)	Type II Clear Cell Carcinoma (<5%)	Type II Carcinosarcoma (<5%)	Type I Endometrioid Carcinoma (±80%)
*TP53*	57.7–92% [34,42,43,44,45,46]	29–46% [47,48,49,50]	64.3–91% [17,42]	10.1–14% [34,42,51]
*PPP2R1A*	15.4–43.2% [34,42,43,44,45,46,52,53,54]	15.9–36% [47,48,49,50,55]	0–28.1% [17,42,54,56,57]	2.5–6.9% [34,42,52,53,54]
*FBXW7*	17.3–29% [34,43,44,45]	7.9–25% [43,48,49,50,55]	39% [17]	10–12% [42,51]
*PTEN*	2.7–22.5% [34,42,45,46]	11–21% [50,55,58]	19–33.3% [17,42]	67–84% [34,42]
*ARID1A*	0–10.8% [34,42]	15–21% [48,49,50,55]	12–23.8% [17,42]	40–46.7% [42,59]
*PIK3CA*	10–47% [34,42,43,44,45,46]	23.8–36% [48,49,50,55]	17–35% [17,42,60]	38–55% [42,51]
*CTNNB1*	2.7% [42]	0% [47,48]	4.8% [42]	23.8–52% [34,42,48,51]
*KRAS*	2–8% [42,51]	12–16.7% [17,42]	14% [17]	16.6–26% [42,51]
*HER2*	17–44% [34,45,61,62,63]	12–50% [61,62,63,64]	0–20% [61,65]	1.4–30% [62,66]

## Abbreviations

4EBP1	Eukaryotic translation initiation factor 4E-binding protein 1
ALK	Anaplastic lymphoma kinase
Bad	Bcl-2-associated death promotor
CIP2A	Cancerous inhibitor of PP2A
CML	Chronic myeloid leukaemia
CT	Computed tomography
EC	Endometrial carcinoma
EGFR	Epidermal growth factor receptor
ERK	Extracellular signal-regulated kinase
FDA	US Food and Drug Administration
FIGO	International Federation of Obstetrics and Gynaecology
FOXO	Forkhead box
GSK-3β	Glycogen synthase kinase 3β
HER-2	Human epidermal growth factor receptor 2
IRS-1	Insulin receptor substrate 1
MAPK	Mitogen-activated protein kinase
MEK	Mitogen-activated protein kinase kinase
MRI	Magnetic resonance imaging
MSI	Microsatellite instability
mTOR	Mammalian target of rapamycin
NSCLC	Non-small cell lung cancer
OA	Okadaic acid
Pap	Papanicolaou
PI3K	Phosphatidylinositol-4,5-bisphosphate 3-kinase
PIK3IP1	PI3K interacting protein 1
PIP_2_	Phosphatidylinositol 4,5-bisphosphate
PIP_3_	Phosphatidylinositol 3,4,5-trisphosphate
PME-1	PP2A methylesterase 1
POLE	Polymerase ε
PP2A	Protein phosphatase 2A
ProMisE	Proactive Molecular Risk Classifier for Endometrial Cancer
PTEN	Phosphatase and tensin homolog
PTPA	Phosphatase 2A phosphatase activator
RTK	Receptor tyrosine kinase
S6	Ribosomal protein S6
SCF	Skp, Cullin, F-box
SET	Suvar/Enhancer of zeste/Trithorax
SMAP	Small molecular activator of PP2A
SV40	Simian virus 40
T-ALL	T-cell acute lymphoblastic leukaemia
TCGA	The Cancer Genome Atlas
TVU	Transvaginal ultrasound
